# Fear conditioning-related changes in cerebellar Purkinje cell activities in goldfish

**DOI:** 10.1186/1744-9081-8-52

**Published:** 2012-10-31

**Authors:** Masayuki Yoshida, Hiroki Kondo

**Affiliations:** 1 Graduate School of Biosphere Science, Hiroshima University, Higashihiroshima, 739-8528, Japan

**Keywords:** Cerebellum, Fear conditioning, Goldfish, Purkinje cell

## Abstract

**Background:**

Fear conditioning-induced changes in cerebellar Purkinje cell responses to a conditioned stimulus have been reported in rabbits. It has been suggested that synaptic long-term potentiation and the resulting increases in firing rates of Purkinje cells are related to the acquisition of conditioned fear in mammals. However, Purkinje cell activities during acquisition of conditioned fear have not been analysed, and changes in Purkinje cell activities throughout the development of conditioned fear have not yet been investigated. In the present study, we tracked Purkinje cell activities throughout a fear conditioning procedure and aimed to elucidate further how cerebellar circuits function during the acquisition and expression of conditioned fear.

**Methods:**

Activities of single Purkinje cells in the corpus cerebelli were tracked throughout a classical fear conditioning procedure in goldfish. A delayed conditioning paradigm was used with cardiac deceleration as the conditioned response. Conditioning-related changes of Purkinje cell responses to a conditioned stimulus and unconditioned stimulus were examined.

**Results:**

The majority of Purkinje cells sampled responded to the conditioned stimulus by either increasing or decreasing their firing rates before training. Although there were various types of conditioning-related changes in Purkinje cells, more than half of the cells showed suppressed activities in response to the conditioned stimulus after acquisition of conditioned fear. Purkinje cells that showed unconditioned stimulus-coupled complex-spike firings also exhibited conditioning-related suppression of simple-spike responses to the conditioned stimulus. A small number of Purkinje cells showed increased excitatory responses in the acquisition sessions. We found that the magnitudes of changes in the firing frequencies of some Purkinje cells in response to the conditioned stimulus correlated with the magnitudes of the conditioned responses on a trial-to-trial basis.

**Conclusions:**

These results demonstrate that Purkinje cells in the corpus cerebelli of goldfish show fear conditioning-related changes in response to a stimulus that had been emotionally neutral prior to conditioning. Unconditioned stimulus-induced climbing fibre inputs to the Purkinje cells may be involved in mediating these plastic changes.

## Background

The cerebellum is important for classical fear conditioning in both mammals and teleost fish. Lesions of certain parts of the cerebellum severely impair changes in heart rate that are induced by conditioned fear in various vertebrate species, including rabbits, rats, and goldfish [1-4]. In goldfish, reversible inactivation of neural activities in the corpus cerebella, through the usage of localized cooling and local administration of anaesthetic agents, suppress the development of classically conditioned fear responses [3,5].

The cerebella in mammals and fish share both a basic histological architecture and neural circuits [6-11]. In addition, it is suggested that Purkinje cells (PCs) have similar synaptic plasticity mechanisms in both mammals and fish [12]. Furthermore, PCs play key roles in cerebellar functions. Thus, investigation of PC functions during classical fear conditioning should further the understanding of the neural mechanisms underlying fear learning and other emotional functions of the cerebellum.

Fear conditioning-induced changes in PC responses to a conditioned stimulus (CS) have previously been reported in rabbits [13]. In that study, differential responses of PCs in the anterior cerebellar vermis to a reinforced conditioned tone stimulus and an unreinforced tone stimulus were observed in animals that had been trained. The cerebellar vermis in mammals has been suggested to be homologous to the corpus cerebelli in teleost fish [14]. Furthermore, it has been suggested that increases in the firing rates [13] and synaptic long-term potentiation (LTP) [15,16] of the PCs are related to the acquisition of a conditioned heart rate change in mammals. This LTP has been suggested to occur at parallel fibre-Purkinje cell synapses as a result of a conjunctive activation of two separate parallel fibre channels, which are activated by CS and unconditioned stimulus (US), converging a single PC [15,16]. Although the increase in the efficacy of parallel fibre-Purkinje cell excitatory synapses was also predicted by Marr’s first theoretical proposal of the functioning of cerebellar circuit, he hypothesized that the potentiation is induced by climbing-fibre input temporally adjacent to the parallel fibre input [17].

However, in the previous study in rabbits, the PC activities were not analysed during the acquisition sessions due to technical difficulties [13]. Thus, while plastic changes in PC synapses induced by fear conditioning have been reported in mammalian species, little is known about changes in the behaviour of PCs *in vivo* during the course of acquisition of conditioned fear.

In the case for discrete motor learning such as classical eyeblink conditioning, contrary to fear conditioning, it was found that PC frequencies during the CS decrease throughout the course of the conditioning procedure [18,19]. Albus [20] postulated in his theoretical work that the synchronous activation of inputs from parallel fibre and climbing fibre to Purkinje cell could result in a long-term depression of the parallel fibre synapses and reducing the Purkinje cell output. Experimental results in the discrete motor learning support this hypothesis: an unconditioned stimulus (US) is conveyed to the Purkinje cell via the climbing fibres, whereas the conditioned stimulus is conveyed by mossy fibres and relayed to the PC via granule cells and then parallel fibres [21,22].

In the present study, we tracked single PC activities throughout the course of classical fear conditioning in goldfish. The fear conditioning paradigm used in the present study was a classical heart-rate conditioning, in which a light was used as conditioned stimulus and electric shock was as aversive unconditioned stimulus. In mammals, control of cardiac activity by the cerebellar vermis has been shown [23,24]. On the other hand, cerebellar control of the cardiac activity in fish has been little known. However, in goldfish, the corpus cerebelli is not essential for the cardiac regulation in response to conditioning-independent simple visual and nociceptive stimuli, while an inactivation of the corpus cerebelli greatly suppress the conditioned cardiac responses [3,5]. We examined learning-related changes in the PC responses to the CS and aimed to further elucidate how cerebellar circuits function during classical fear conditioning.

## Methods

### Subjects

Goldfish (*Carassius auratus*), 73–92 mm in standard length, were commercially obtained and kept in our laboratory at a water temperature of 23–26 °C with a photoperiod of 14 h light/10 h dark. The fish were maintained in these conditions for more than 3 weeks before they were subjected to experiments. Experiments were performed during the light period. All animal experiments were conducted in accordance with the Guidelines for Animal Experimentation, Hiroshima University.

### Classical fear conditioning and neuronal recording

Goldfish were anaesthetised in 0.015% tricaine methanesulfonate (Crescent Research Chemicals, Phoenix, AZ, USA), and then d-tubocurarine chloride (Nacalai Tesque, Tokyo, Japan) (5 μg/g body weight) dissolved in phosphate-buffered saline was intraperitoneally injected to immobilise the fish. A window (5 × 5 mm) was opened in the cranium, and the dorsal surface of the corpus cerebelli was exposed by removing the fat deposits beneath the cranium. The fish were placed in a conditioning chamber and gently restrained between a pair of urethane holders. The gills were irrigated with aerated water through a tube that was inserted into the mouth. Water levels in the chamber were adjusted so that the window in the cranium remained slightly above the water surface, and then 3–5% low-gelling-temperature agar (Nacalai Tesque, Tokyo, Japan) in saline was poured into the cranial cavity to stabilise the electrode that was subsequently inserted into the corpus cerebelli.

The goldfish were allowed to recover from the anaesthesia for about 1 h, and then the activities of the cerebellar neurons were recorded. Recordings were made from one PC per fish, and 21 recordings were subjected to analysis. Although 77 goldfish were used in the experiment, data from 56 fish were not subjected to further analysis due to insufficient recording quality, loss of the unit before the completion of conditioning procedure or ambiguities in the identity of recorded neurons. Glass capillary electrodes (10 MΩ), tungsten wire electrodes (0.8 MΩ), or stainless steel wire electrodes (1 MΩ) were used for extracellular unit recordings. No substantial differences in the quality of the recordings were observed among these three types of electrodes. Recordings were made in the dorsal portion of the corpus cerebelli. Since it is not known which subdivisions of the corpus cerebelli are involved in classical fear conditioning, the recording site locations were reported as follows: 1) rostral and ipsilateral to the CS-presented side; 2) rostral and contralateral to the CS-presented side; 3) caudal and ipsilateral to the CS-presented side; and 4) caudal and contralateral to the CS-presented side. PCs were identified according to previous studies on the basis of types of action potentials (simple or complex spikes) and the depths (500–800 μm) of the recording positions [25,26].

Classical heart rate conditioning was performed using the delay conditioning paradigm. The CS was a 5.1 s illumination of a red light-emitting diode (LED) that had been placed on either side of the head. The unconditioned stimulus (US) was a 10–20 V electric shock to the trunk delivered via a pair of silver plates (10 × 10 mm). The conditioning procedure consisted of habituation (six to ten trials), acquisition (20–25 trials), and extinction sessions (15–20 trials). In the habituation and extinction sessions, only the CS was presented. In the acquisition sessions, the US was presented 5 s after the onset of the CS. The intertrial interval was 60 s for all sessions.

The conditioned responses were decelerations of heartbeats or bradycardias during the presentations of the CS. The heartbeats were noninvasively recorded using a photocardiography technique [27]. Since the conditioned bradycardic responses are the most apparent responses observed immediately after the onset of the CS [3], the magnitudes of the cardiac responses to the CS were quantified as follows. First subtracting the average heartbeat frequencies during the first 2 s of the CS presentations from the average heartbeat frequencies during a 2 s period prior to the onset of the CS (pre-CS heartbeat frequencies). The obtained values were then divided by the pre-CS heartbeat frequencies. If no heartbeat occurred during the first 2 s of the CS, the magnitude value was 1. If tachycardia occurred in response to CS, the value was a negative value. To compare the levels of acquisition among individuals, the average conditioned responses in the last ten trials of the acquisition sessions were also calculated as the conditioned scores.

### Analysis of PC activity

Off-line analyses of PC activities were performed using the software package, LabChart (AD Instruments, Sydney, NSW, Australia). The 10 s period of each trial, including the 2 s pre-CS period, was divided into ten 1 s bins to analyse the PC responses. The firing frequencies of separated simple spikes were calculated for each bin and then averaged over each session. For acquisition sessions, the values of the first and last ten trials were separately calculated. Firing frequencies of simple spikes during the CS were compared with those observed prior to the CS onset to analyse the types of PC responses to the CS presentations. In cases in which the bin with the highest firing frequency during CS was significantly larger than the bin with the highest frequency in the pre-CS 2 s (Wilcoxon signed rank test, p < 0.05), the PC was classified as CS-excited. In cases in which the bin with the lowest frequency during CS was significantly smaller than the bin with the lowest frequency in the pre-CS 2 s (Wilcoxon signed rank test, p < 0.05), the PC was classified as CS-inhibited. In cases in which enhanced responses of the CS-excited or CS-inhibited PCs to the CS was observed during acquisition session, Mann-Whitney U test was used to compare average response magnitude in habituation session with that in the acquisition session.

In goldfish, firing frequencies of the complex spikes were low (usually less than 1 Hz) [25] and artifacts due to the electric shock US were considerably large. This situation prevented us with accurate peristimulus analysis of complex spike firings in some cases. Perievent analyses of complex spikes relative to the US were only performed for 11 PCs in which electric shock artifacts were sufficiently small

All statistical analyses were performed using the R software package. Differences were considered significant when p < 0.05.

## Results

### Conditioning

**Figure 1:**
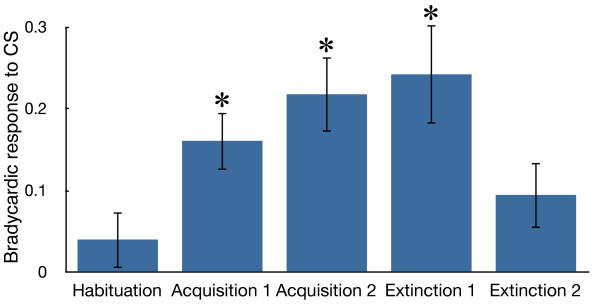
**Classical fear conditioning results.** Comparison of the magnitudes (mean ± SEM) of the bradycardic responses in the habituation sessions, the first ten trials of the acquisition sessions (Acquisition 1), the last ten trials of the acquisition sessions (Acquisition 2), the first five trials of the extinction sessions (Extinction 1), and the last ten trials of the extinction sessions (Extinction 2). * denote significant differences relative to the habituation levels (Steel test, p < 0.05).

**Figure 2:**
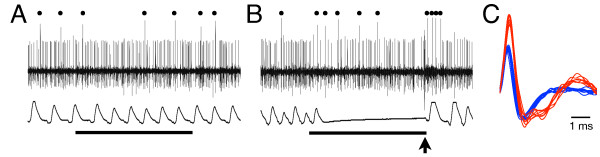
**Simultaneous recordings of PC activities and heartbeats during the conditioning procedure.A**. The eighth trial in the habituation session. **B**. The 21st trial in the acquisition session. In this case, firing frequency of simple spikes was decreased during the first two seconds of the CS presentation in the 21st trial in the acquisition session (B). PC activities and heartbeats are shown in upper traces and lower traces, respectively. Dots indicate complex spikes. Horizontal bars indicate 5.1 s periods of CS presentations. The arrow indicates the timing of the US presentation. **C**. Superimposed recordings of simple (blue) and complex (red) spikes that were observed in A.

Figure 1 shows a comparison of the magnitudes of the bradycardic responses to the CS observed in the five stages of the conditioning procedure used in this study. The fish tended to respond to the first presentation of the CS in the habituation session with cardiac deceleration. This response was quickly habituated within a few trials as reported previously [3]. Conditioned bradycardic responses developed during an early stage of the acquisition session, and the differences from the habituation levels were statistically significant for both the first ten acquisition trials (acquisition 1) and the later stage of the acquisition sessions (acquisition 2) (Steel test, p < 0.05). The conditioned response was not timed to the US but usually occurred immediately after the onset of the CS, and in extreme cases, the heartbeat was almost absent until the end of the CS (see Figure 2B). This observation was consistent with the previous reports [3,5] and characteristic to the present cardiac conditioning system of the goldfish. The conditioned responses were significant in the early trials of the extinction sessions (extinction 1), in which the CS were not accompanied by the US (Figure 1). These data confirm that CS-induced bradycardias were anticipatory fear responses. In the latter phase of the extinction sessions, the conditioned responses were reduced to levels that did not differ significantly from the habituation levels. 

### Simple-spike response of PCs

Among 21 PCs that were traced throughout the conditioning procedure, 12 showed simple-spike firing frequencies that were changed by the CS during the habituation sessions. Table 1 shows a summary of the PC responses to the CS and the changes in these responses after the acquisition trials, and Figure 2 shows actual examples of simultaneous recordings of PCs and heartbeats. Eight PCs showed excitatory responses and four PCs showed inhibitory responses to the CS before the acquisition sessions. There were no significant differences in the conditioning scores among the fish with CS-excited PCs, CS-inhibited PCs, and PCs that showed no response to the CS in the habituation sessions (Steel-Dwass test, p > 0.05).

**Table 1:** Responses of the PCs to the CS and the patterns of conditioning-related changes

**Number of responses to the CS in habituation sessions**		**Number of responses to the CS in the last ten trials in the acquisition sessions**	
excitatory	8	increased excitation	1
		decreased excitation	1
		inhibition (reversed)	5
		no change	1
inhibitory	4	increased inhibition	1
		excitation (reversed)	1
		no change	2
no response	9	excitation	1
		inhibition	4
		no change	4

**Figure 3:**
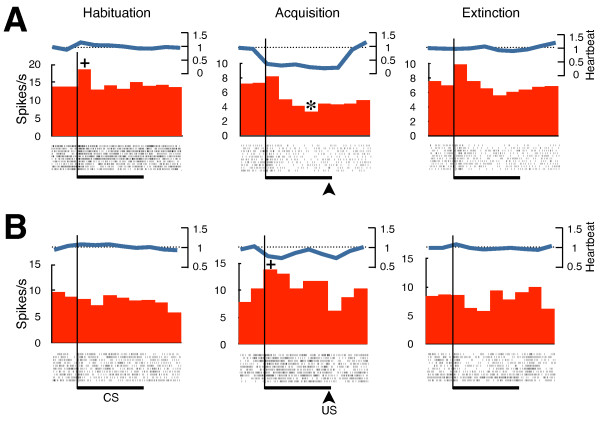
**Peristimulus time histograms of simple spikes and heartbeat frequencies in the habituation, acquisition (last ten trials), and extinction sessions.** Average responses of ten consecutive trials in each session are shown. Relative heartbeat frequencies in relation to average heartbeat frequency in the 2 s before the CS are shown. Rastergrams of simple-spike timings are also shown below the frequency plots. Horizontal bars indicate periods of CS presentations. Arrowheads indicate the timing of the US presentations. + and * denote significantly higher and lower frequencies relative to the highest or lowest bin in the 2 s before the CS, respectively (Wilcoxon signed rank test, p < 0.05). CS: conditioned stimulus; US: unconditioned stimulus; s: seconds.

Fourteen PCs exhibited changes in the response magnitudes or patterns to the CS in the last ten trials of the acquisition sessions. One PC showed a significant increase (Mann-Whitney U test, p < 0.05) of the excitatory response to the CS and one showed decreased excitatory response. Five PCs that showed excitatory responses to the CS during the habituation sessions reversed the direction of the response after acquisition of the conditioned responses (Figure 3A). They exhibited significant suppression of the firing rates (Wilcoxon signed rank test, p < 0.05) during the CS in the last 10 trials of the acquisition session.

We observed four PCs that responded to the CS with suppressed firing rates in the habituation sessions. One of these PCs showed increased inhibitory responses to the CS after acquisition of the conditioned responses (Mann-Whitney U test, p < 0.05). The response pattern in one PC reversed from inhibitory to excitatory (Wilcoxon singed rank test, p < 0.05). The other two PCs showed no significant changes after repeated paired presentations of the CS and US, even though the conditioning scores of the goldfish from which these PCs were recorded were significantly greater than zero (Mann-Whitney U test, p < 0.01).

Five PCs that showed no responses to the CS during the habituation sessions responded to the CS either with significantly increased (n = 1) (Figure 3B) or decreased (n = 4) firing rates during the acquisition sessions (Wilcoxon signed rank test, p < 0.05).

In the three PCs that showed excitatory changes in the responses to the CS in the conditioned states when compared with the responses in the habituation sessions, the firing frequencies of the first bins after the onset of the CS presentations were the highest. However, in the PCs that showed inhibitory changes after acquisition in response to the CS, the bins with the lowest firing frequencies differed from fish to fish. In these cases, the lowest firing frequencies were observed in the following bins: in the first bin (n = 1); in the second bin (n = 3); in the third bin (n = 1); in the fourth bin (n = 3); and in the fifth bin (n = 2). We observed no apparent relationships between the positions of the PCs in the dorsal corpus cerebelli and the patterns of responses to the CS.

**Figure 4:**
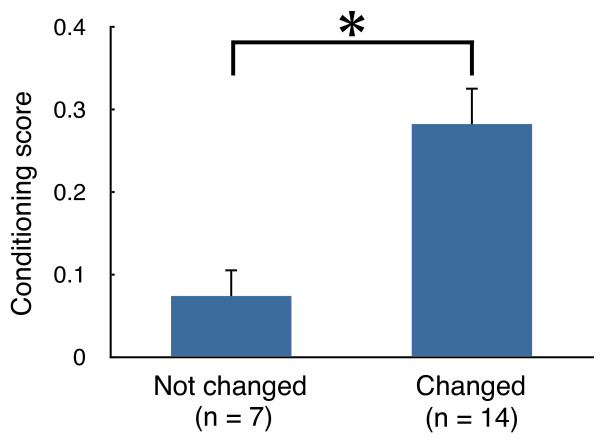
**Comparison of the conditioning scores between fish with different PC responses to the CS.** Conditioning scores (mean + SEM) for the fish with PCs that showed conditioning-related changes in response to the CS were greater than the scores in the fish with PCs that did not show conditioning-related changes in the response to the CS. Conditioning scores were calculated by averaging the conditioned responses in the last ten trials of the acquisition sessions. * denotes a significant difference (Mann-Whitney U test, p < 0.01).

The conditioning scores of the goldfish in which PCs showed changes in the responses to the CS in the acquisition sessions relative to the habituation sessions (n = 14) were greater than the conditioning scores of the fish in which PCs showed no changes in response to the CS during the conditioning procedure (n = 7) (Mann-Whitney U test, p < 0.01) (Figure 4).

In the extinction sessions where only the CS was presented, the response patterns of the PCs were similar to those observed in the habituation sessions (Figure 3). These data indicated that the patterns of changes in response to the CS after acquisition of the conditioned responses were correlated with the states of conditioned fear.

**Figure 5:**
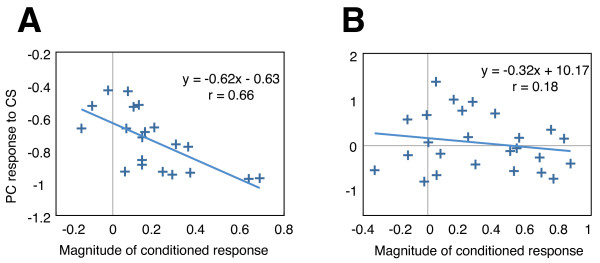
**Relationship between magnitudes of conditioned responses and PC responses during the first 2 s of CS presentations in the acquisition sessions.A**. Representative data from a fish that showed an significant correlation (p = 0.002) between the conditioned response magnitude and the level of suppression of PC activity during the CS. **B**. Representative data from a fish that did not show a correlation between the magnitude of the conditioned response and the response of the PC to the CS. The vertical axes show relative spike frequencies of the PCs relative to the frequencies in the 2 s before the CS. Inhibitory responses of the PCs to the CS were assigned negative values. Formulae of the linear regressions and r-values were also shown. PC: Purkinje cell; CS: conditioned stimulus.

To reveal if there were any relationships between the PC responses to the CS and conditioned bradycardic responses, we examined the correlations between the magnitudes of the PC responses to the CS and the magnitude of the conditioned bradycardias in the acquisition sessions. We found that the magnitudes of the changes in firing frequencies of the six PCs in response to the CS were significantly correlated (p < 0.05) to the magnitudes of the conditioned responses across a number of different trials (Figure 5A). In all of these cases, either greater inhibitory or smaller excitatory responses were observed in the trials where greater bradycardic responses were evoked by CS presentations. In addition, these six PCs were not localised to certain portions of the corpus cerebelli, but rather were distributed in all quarters of the corpus cerebelli (rostral, caudal, and ipsilateral and contralateral to the CS-presented side). In the other 15 PCs that were monitored, there were no apparent correlations between PC response magnitudes and conditioned cardiac responses (Figure 5B).

There were no correlations observed between heartbeat frequencies and firing frequencies of the PCs in the same 1 s bins or time windows (r < 0.2 in all cases), which indicated that the recorded PCs were not involved in direct control of heartbeats.

### Complex-spike response of PCs

**Figure 6:**
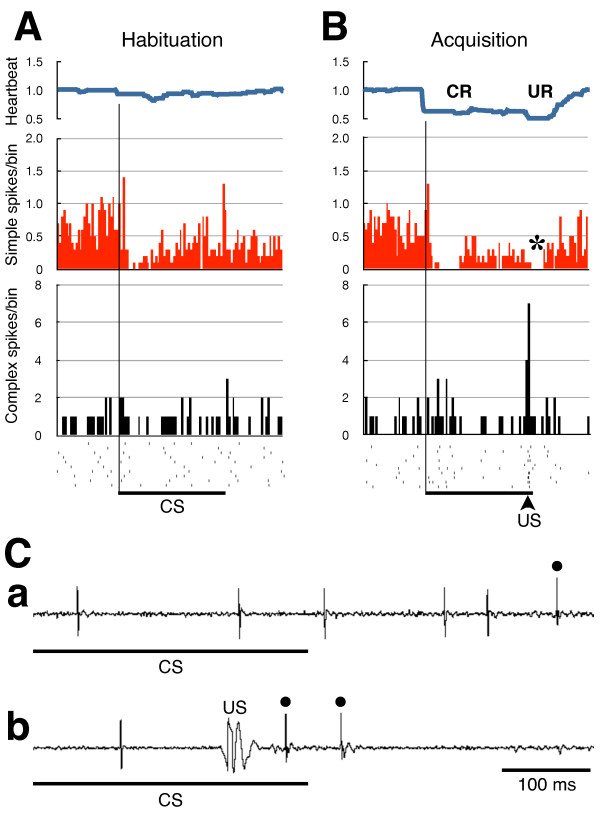
**Conditioning-related changes in the simple-spike responses to the CS, and US-evoked complex-spike firings in a PC.** Average responses of ten consecutive trials in a habituation session (**A**) and an acquisition session (**B**), respectively. Relative heartbeat frequencies relative to the average heartbeat frequencies in the 3 s before the CS are shown. Rastergrams of complex-spike timings are also shown at the bottom. CR: conditioned response; UR: unconditioned response. * denotes a pause in simple-spike firing after the US presentation. The arrowhead indicates the timing of the US presentation. Bin width = 100 ms. **C**. Representative recordings at the end of the CS in habituation (**a**) and acquisition (**b**) sessions. In acquisition session (b), complex spikes (dots) were evoked by US with relatively short latencies. CS: conditioned stimulus; US: unconditioned stimulus. ***A***, **B** and **C** were obtained from the same preparation.

Among the 11 PCs with electric shock artefacts that were small enough so that peristimulus complex-spike firings could be analysed, seven responded to the US with complex-spike firings (Figure 6). The other four PCs did not show complex-spike firings that were time locked to US presentations. In three of the seven PCs, transient pauses in firings of simple spikes after US presentations were also observed (Figure 6B,C). In addition, the majority (six out of seven) of the PCs that showed US-coupled complex-spike firings exhibited conditioning-related inhibitory changes in simple-spike responses to the CS. One PC did not show conditioning-related changes in response to the CS. Peak latencies of the complex spike response in relation to the US varied among fish and typically ranged between 30-150 ms except for one case in which the latency was about 350 ms. We didn’t find any tendency that the PCs with shorter latency of the complex spike response to the US showed greater change in simple spike activity after acquisition of the conditioned response.

We found that the PCs that did not show US-coupled complex-spike firings tended to show conditioning-related increases in simple-spike firing frequencies upon CS presentations. We also found that, among the PCs which showed complex-spike firings in response to US, only two PCs’ simple spike activity was significantly correlated with the magnitude of the conditioned responses across a number of different trials.

## Discussion

Fear conditioning is essential for survival and is characterized by the rapid acquisition and long-term persistence of a fear response [15,28]. The fear-related heart rate conditioning identified in the present study is not unusual. While eyeblink conditioning typically requires dozens to hundreds of paired stimulations of CS and US to obtain sufficient conditioned responses [4,29-31], the conditioned bradycardic responses in fear conditioning in goldfish reach an asymptotic level within only 15 to 20 trials of paired stimulations [3,5].

In mammals, the cerebellar hemisphere and the vermis are essential for eyeblink conditioning and heart rate conditioning, respectively [1,32,33]. Furthermore, it is suggested that the corpus cerebelli in teleost fish is homologous to the vermal portion of the mammalian cerebellum [14]. In goldfish, lesions of the corpus cerebelli impair both eye-retraction conditioning, which is thought to be analogous to eyeblink conditioning [4,31], and emotional heart rate conditioning [3,4]. However, whether the corpus cerebelli in teleost fish plays roles homologous to both the vermal and hemispheric portions of the mammalian cerebellum remains unknown.

The present study aimed to examine learning-related changes in the PC responses to the CS and to further elucidate how cerebellar circuits function during classical fear conditioning. We successfully tracked single PC activities throughout the conditioning procedures, which enabled examination of the relationships between the levels of conditioned response and the activities of PCs throughout acquisition of conditioned responses. Fear conditioning-related changes in PC activities have also been reported in rabbits [13]. However, no comparisons were performed between the responses of single PCs to the tone CS before and after acquisition of the conditioned bradycardic responses because of technical difficulties. Therefore, the authors used a differential conditioning procedure with two different tones, one that was reinforced (CS+) and one that was unreinforced (CS-). The differential responses to CS+ and CS- were then analysed to evaluate the conditioning-related PC responses. In the present study, we were able to observe the activities of single PCs throughout the conditioning procedure, which enabled direct comparisons of the responses of the PCs to the CS between naive and conditioned states with a relatively simple conditioning procedure.

More than half of the PCs sampled in the present study responded to the CS with either increased or decreased simple-spike firing rates in the habituation sessions where the CS was not accompanied by the US, indicating that LED light illumination activated multiple pathways originating from the retina to the corpus cerebelli. After acquisition of the conditioned fear responses, one CS-excited PC showed a greater excitatory response to the CS relative to the response before conditioning. By contrast, five CS-excited PCs reversed their pattern of the response to the CS relative to the response before conditioning. These observations suggest that, in PCs of which simple-spike responses were suppressed after training, excitatory parallel fibre inputs to the PC induced by CS were weakened after acquisition of conditioned response. In addition, since the firing frequency in these PCs during CS was below the level of that before the CS, it is possible that inhibitory inputs from cerebellar interneurons were increased at the state of conditioned fear. Similarly, of the four CS-inhibited PCs in the habituation sessions, one showed increased inhibition, and one showed a reversed response to the CS after acquisition of conditioned fear. Nine PCs showed no responses to the CS before conditioning. However, among these cells, one PC showed excitatory, and four showed inhibitory responses to the CS in the last ten trials of the acquisition sessions.

Although a relatively small number of PCs showed increased excitatory responses to the CS as the acquisition sessions proceeded, the short latencies of the increased firing rates suggest that these neurons might be involved in autonomic fear responses, including conditioned bradycardias. However, as no positive or negative correlations were observed between heartbeat and firing frequencies of the PCs in the same 1 s time window, it is unlikely that the PCs recorded in the present experiments directly controlled the heart regulation centre.

On the other hand, more than half (79%) of the PCs that showed conditioning-related changes in simple-spike response to the CS also showed increased inhibitory responses after acquisition of conditioned fear. These conditioning-related suppressions of PC responses to sensory inputs caused by the CS might be induced by climbing fibre inputs that came just after the US at the end of the CS (see Figure 6). This observation supports Albus’ theory of plastic changes in the cerebellar circuitry [34], in which simple-spike firing in response to the CS is suppressed after CS-US pairings. Therefore, it is possible that US-induced climbing fibre inputs to the PCs play an important role in the plastic changes seen in PCs with classical fear conditioning. However, the present results were not fully consistent with Albus’ theory. Among the seven PCs that showed US-evoked complex-spike firings, only three exhibited US-induced pauses in simple-spike firing. Albus postulated this behaviour as a central feature of the cerebellar circuitry. In the present study, we were able to perform analyses of US-related complex-spike firings of PCs, which reflect climbing fibre inputs, in only about half of the fish due to technical limitations. In future studies, artefact-free US presentations must be developed so that further investigations into the mechanisms underlying the plasticity of the cerebellar circuitry in fear conditioning can be performed.

In the previous study that looked at PC responses in fear-conditioned rabbits, 52% of recorded PCs were reported to show differential responses to the CS+, which was paired with the US, relative to the CS-, which was not paired with the US [13]. Furthermore, in contrast to the results seen here, most of the PCs (> 86%) in this study showed differential increases in firing frequencies in response to the CS+, while only a small number of PCs showed differential decreases in activity in response to the CS+. These multiple types of conditioning-related changes in PC responses to the CS have also been reported with classical conditioning of the nictitating membrane responses of rabbits [22-24]. Neither Marr [17] nor Albus [20] predicted such diversity of conditioning-related PC responses [34]. Further investigations involving simultaneous multiple PC recordings may help to elucidate the cerebellar circuitry functions that underlie classical conditioning.

The results of the present study demonstrate that the population of PCs in the cerebellum showed multiple response patterns to the CS (see Table 1), and that there were also various fear conditioning-related changes in the response patterns. It is unlikely that the cerebellum is only involved in heart rate conditioning in fear-related learning situations. Projections from the corpus cerebelli have been shown to reach various regions of the brain [35]. The present results demonstrate that the responsiveness of some PCs to the CS correlated with the magnitude of the conditioned bradycardia, suggesting that a part of neuronal population in the corpus cerebelli determine the intensities of conditioned fear responses.

We also observed that behavioural extinction resulted in PC response patterns to the CS that were similar to those patterns observed before acquisition of conditioned fear. This result is consistent with the hypothesis originally suggested for eyeblink conditioning, in which the conditioning can be explained at the level of a single PC [19].

A disadvantage of the present goldfish fear conditioning system is that the relatively low firing frequencies of PCs and the low-basal heart rates made it difficult to perform analyses of neural activities and cardiac changes at higher temporal resolutions. These analyses could have helped to estimate causal relationships. Further investigations are needed to elucidate the possibility that emotional fear conditioning and discrete motor conditioning involve different mechanisms while sharing the same basic cerebellar circuitry.

## Conclusions

The results of the present study demonstrate that PCs in the corpus cerebelli of goldfish show fear conditioning-related changes in response to a stimulus that had been neutral prior to conditioning. US-induced climbing fibre inputs to the PCs is suggested to be involved in mediating these plastic changes. Activities of the neuronal population of the corpus cerebelli are correlated with the levels of conditioned fear.

## Competing interests

The authors declare that they have no competing interests.

## Authors’ contributions

MY designed the study and drafted the manuscript. MY and HK performed the experiments in the study. Both authors read and approved the final manuscript.
